# The difficult management of patients with respiratory segmental Dystonia

**DOI:** 10.1016/S1808-8694(15)31078-8

**Published:** 2015-10-22

**Authors:** Noemi Grigoletto De Biase, Paulo Augusto de Lima Pontes, Vanier Santos Junior, Vanessa Pedrosa Vieira, Priscila Zambonato, Reinaldo Kazuo Yazaki

**Affiliations:** 1 PhD in Medicine - Federal University of São Paulo - Paulista School of Medicine. Visiting Professor - Federal University of São Paulo - Paulista School of Medicine - Associate Professor - School of Hearing and Speech Therapy – -Pontifícia Universidade Católica de São Paulo.; 2 PhD in Medicine - Paulista School of Medicine, 1970 PhD in Medicine - Paulista School of Medicine, 1981. Associate Professor of Otorhinolaryngology - Department of Otorhinolaryngology/Ophthalmology Paulista School of Medicine, 1989. Full Professor - Department of Otorhinolaryngology and Human Communication Disorders - Paulista School of Medicine, 1991; 3 MD. Otorhinolaryngologist - Fundação para o Estudo e Tratamento das Deformidades Crânio-Faciais; FUNCRAF, Santo André; SP.; 4 Postgraduate Student in Internal Medicine and Therapeutics - UNIFESP - Specialization in Human Communications Disorders - UNIFESP - Specializing in Voice - CEV.; 5 MD. Resident Physician in Pediatric Otorhinolaryngology - Department of Otorhinolaryngology and Head and Neck Surgery - Federal University of São Paulo - UNIFESP - EPM.; 6 MD. Resident Physician in Otorhinolaryngology - Department of Otorhinolaryngology and Head and Neck Surgery - Federal University of São Paulo - UNIFESP - EPM

**Keywords:** dyspnea, segmental dystonia, pharynx, larynx, respiration

## Summary

Respiratory dystonia is a rare and difficult to diagnose disorder, that causes breathing restriction of various degrees. The objective of the study is to report the case of a patient with respiratory dystonia involving the larynx and the pharynx and its evolution concerning spasms intensity and control. **Case Report:** A 24 year-old-man has been followed for 5 years. The diagnosis was made by means of nasofibroscopy and electromyography. Treatment was carried out with laryngeal and pharyngeal Botulin toxin injections, as it became necessary for symptoms control. **Conclusion:** The difficult management can be secondary to the lack of knowledge on the etiology and physiopathology of the impairment, and because of the limitations in the treatment of associated respiratory symptoms.

## INTRODUCTION

**D**ystonia is a movement disorder, characterized by involuntary and continuous muscle contractions, such as torsion, repetitive movements or abnormal posture. It may affect any body part, including axial, cranial and limb muscles, causing functional loss. It usually worsens in states of fatigue, stress, emotion and motor activities, and they are definitely not psychogenic affections1. Although it is not a fatal disease, it is chronic and often times of difficult diagnosis and prognosis. These abnormal movements may be kept on for variable amounts of time, from seconds to minutes. Its cause is unknown, although most authors consider basal ganglion involvement^2^.

Among movement disorders, this is the third most common, after Parkinson and tremor. In North America there are over 3 thousand people diagnosed with different types of dystonias and it may affect any ethnical and age group. ^3^.

Dystonia is only recently accepted as a distinct entity. It was Hermann, in 1911, who described muscle tone variations as a neurologic syndrome observed in younger adults. He called this muscle tone variations and spasms Dystonias and described that these movements started after voluntary movements. After this first definition, other efforts have been made in order to better define a dystonia. More recently, the Scientific Advisory Board of the Dystonia Medical Research Foundation submitted a new definition: “Central Motor Processing Disorder, characterized by abnormal involuntary movements, uncontrollable spasms, usually activity-induced”. They also classified dystonias in three types: by age of onset, by affected body part and by etiology. Recently, the progress in genetic research has been changing the etiologic classification by means of describing the genetic code of people affected by such syndrome^3^.

Dystonia is also classified according to the number of body parts or regions involved. It may be half of the body, or it may be generalized - when it involves many body segments. In children, symptoms are of focal onset, followed by generalization to other body parts, while in adults the symptoms usually remain focal^4^.

Dystonia with laryngeal involvement, earlier called spasmodic or spastic, has been recently called focal laryngeal dystonia, and such terminology is more broadly accepted today. Laryngeal focal dystonia bears voice alteration as its major manifestation, caused by spasms or involuntary tremor of the vocal folds that break the voice, and impact not only voice quality, but also speech intelligibility depending on involvement extension.

A less frequently found form of focal laryngeal dystonia is the respiratory type, a more concerning type of dystonia for it causes respiratory distress of varied degrees, that usually disappears with sleep, and does not alter the patient's voice during speech^1^,^5^. On respiratory dystonias, we notice a paradoxical vocal fold movement during inspiration, with early adduction that prevents air intake at the end of inspiration; if with progress, glottic opening time is reduced, there might not be enough vocal fold displacement to allow proper breathing, then there is the need for tracheostomy for proper ventilation. Electromyography is characteristic because it shows the electrical activity of the adducting muscles - the thyroarytenoid and the lateral cricoarytenoid during inspiration.

Vocal therapy, psychotherapy, and a number of other drugs such as benzodiazepines, anticholinergic and dopamine blockers have been used in its treatment. Notwithstanding, the most effective one is Botulin toxin injection in the affected muscles^6^.

We, hereby present a case of segmentary dystonia affecting the larynx, pharynx and palate, with contraction force intensification and the need of repeated treatments with Botulin toxin, a difficult case to manage.

## CASE REPORT

**Figure 1:**
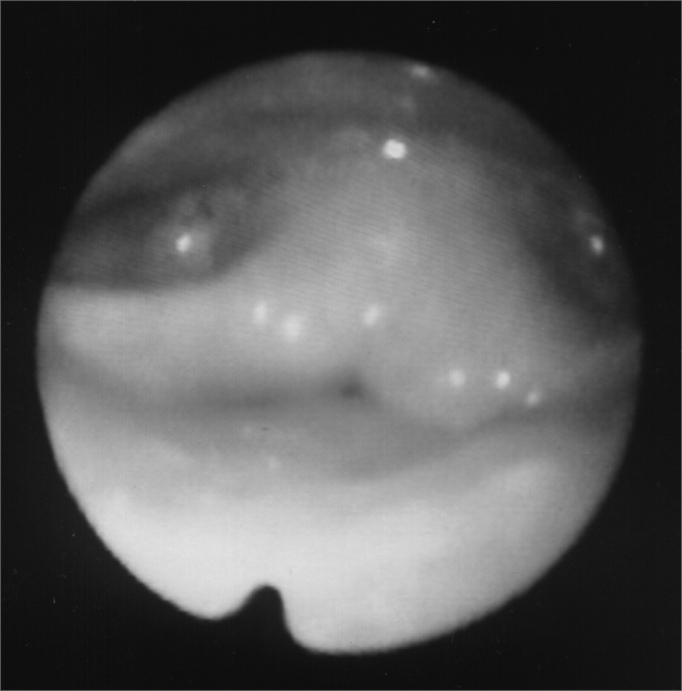
Larynx at the end of inspiration

**Figure 2:**
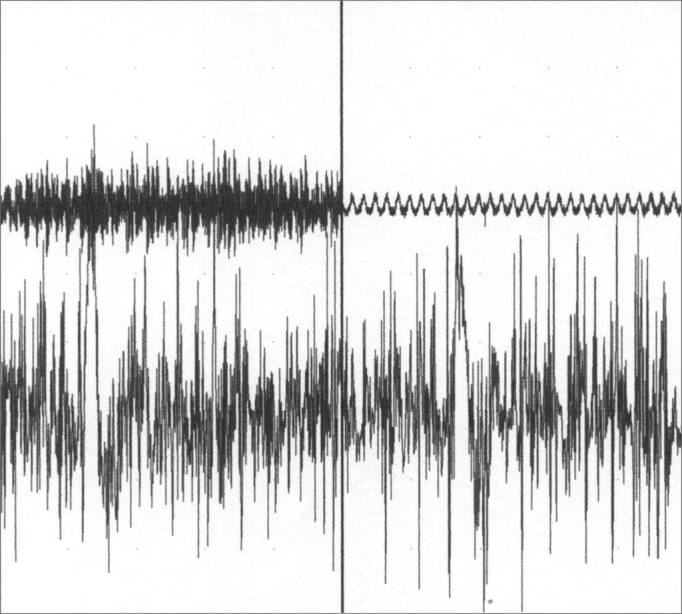
Left thyroarytenoid muscle electromyography. Upper trace: vocal emission sound captured by a microphone. Lower trace: electrical potentials captured from the muscle by means of a concentric monopolar needle. Left image: during phonation. Right side image: during inspiration.

**Figure 3:**
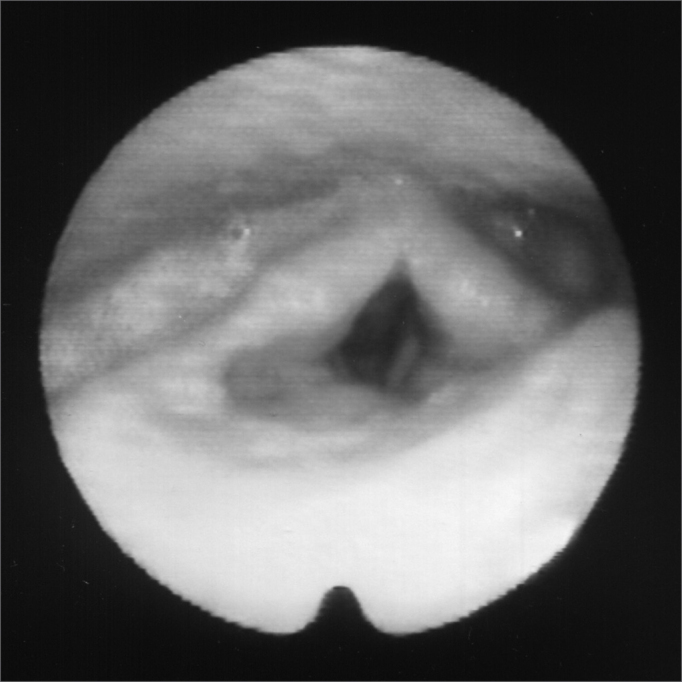
Larynx at rest, after Botox injection

**Figure 4:**
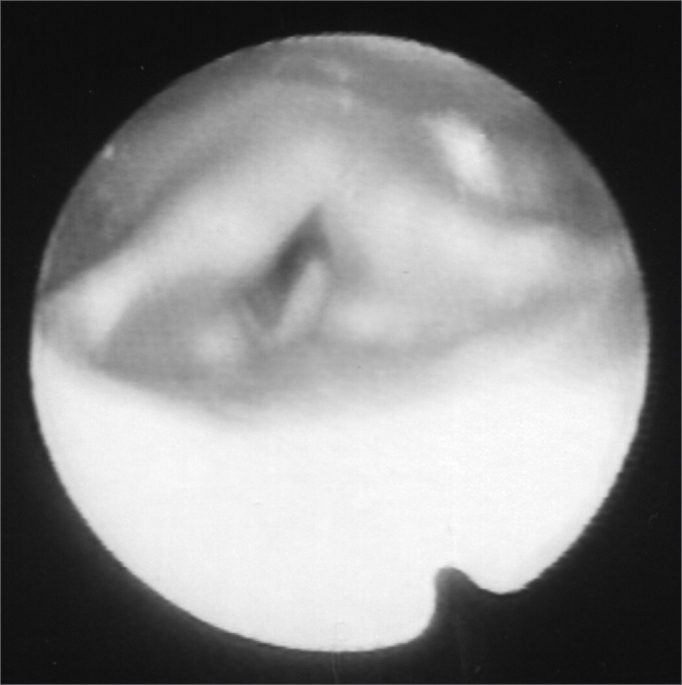
Larynx after Botox injection, during inspiration

**Figure 5:**
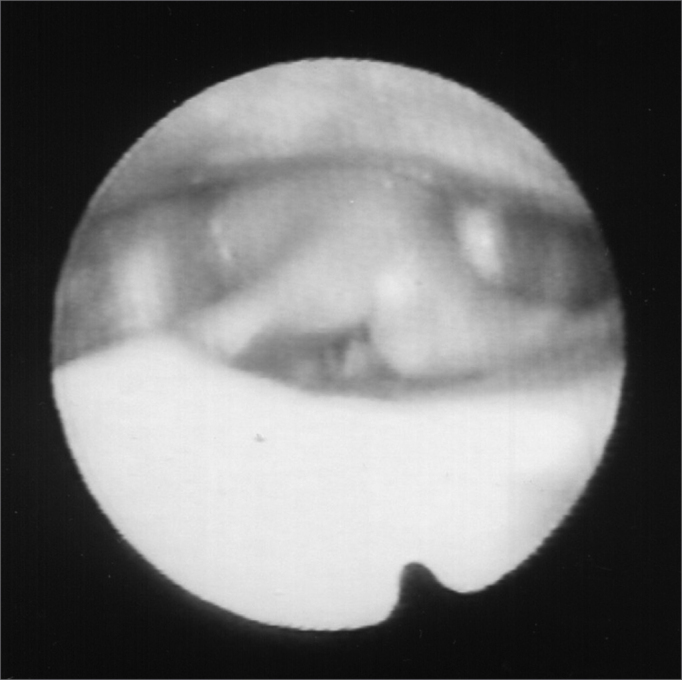
Larynx during phonation after Botox injection

A 24 year old male, Caucasian, with history of dyspnea during deep inspirations for 5 years. He did not have vocal or dysphagia complaints. He reported epiglottis malacia and its partial removal in another service, with little improvement in his dyspnea and having pain during swallowing. During nasopharyngolaryngoscopic exam we observed a tongue base posteriorly positioned, and the patient was submitted to lingual tonsillectomy, without improvement. In a new exam, we noticed a paradoxical laryngeal movement, with glottic closure at the end of inspiration, followed by a slight pharyngeal closure (Figure 1). Larynx electromyography revealed electrical activity in the thyroarytenoid (TA) and lateral crycoarytenoid (CAL) muscles during inspiration (Figure 2). Botulin toxin injection was done on the left TA (10 UI) and left CAL (10 UI) with clinical improvement for 5 months (Figures 3, 4, 5); such injections were repeated and, facing a worsening of his clinical manifestations, it was necessary to inject also the aforementioned muscles of the right side, with temporary improvement and symptoms relapse when the toxin effect was over, requiring new injections.

**Figure 6:**
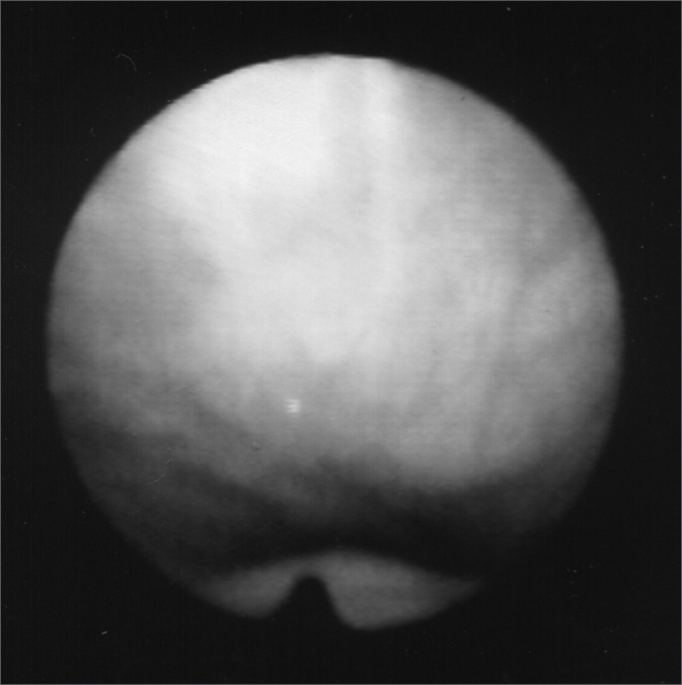
Palate at rest

**Figure 7:**
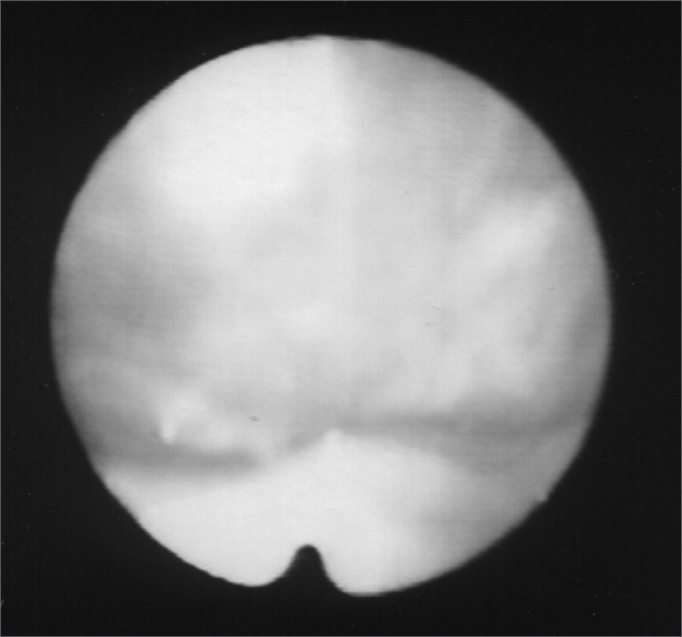
Palate during nasal breathing

**Figure 8:**
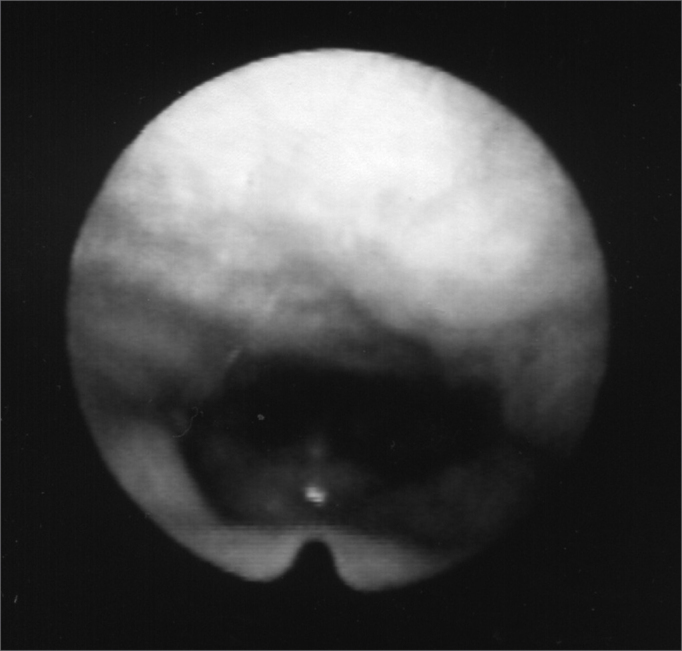
Palate after Botox injection; pharynx at rest

**Figure 9:**
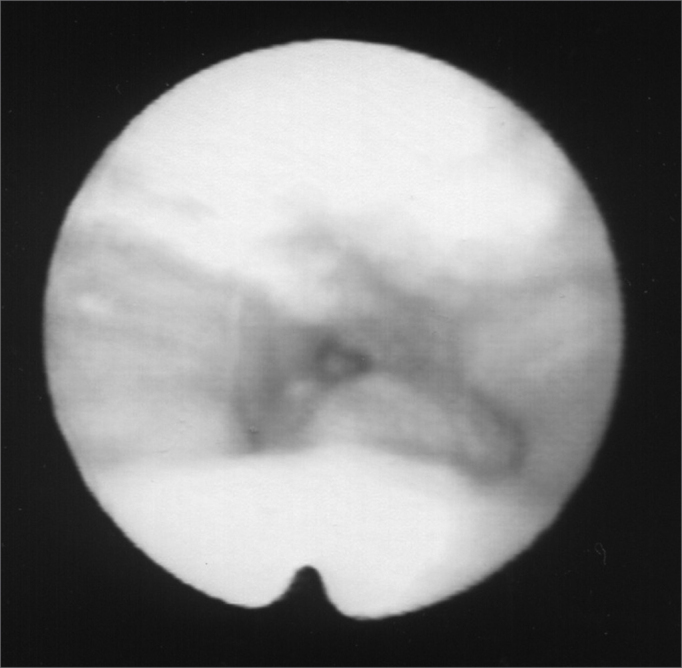
Palate after Botox injection; pharynx contracted during inspiration

**Figure 10:**
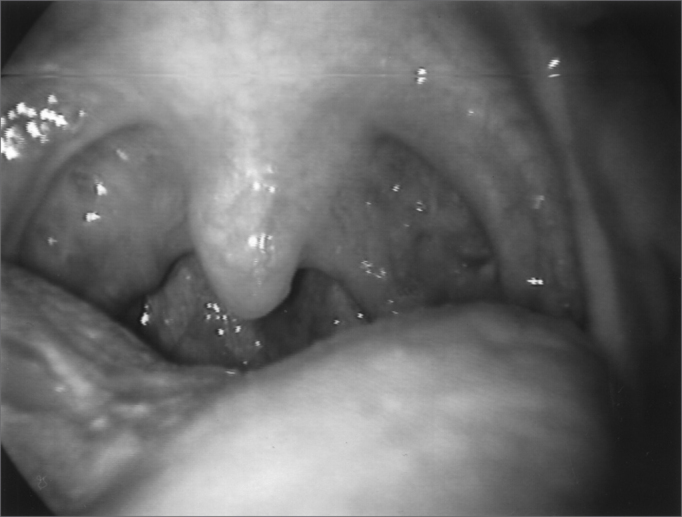
Pharynx at rest

**Figure 11:**
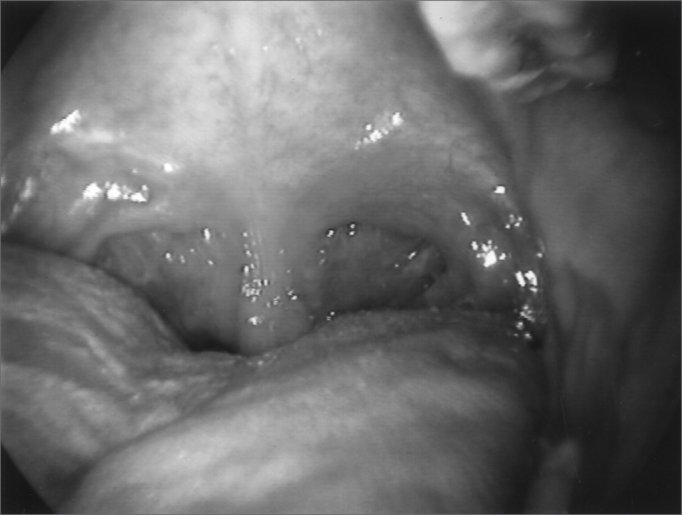
Pharynx during inspiration

**Figure 12:**
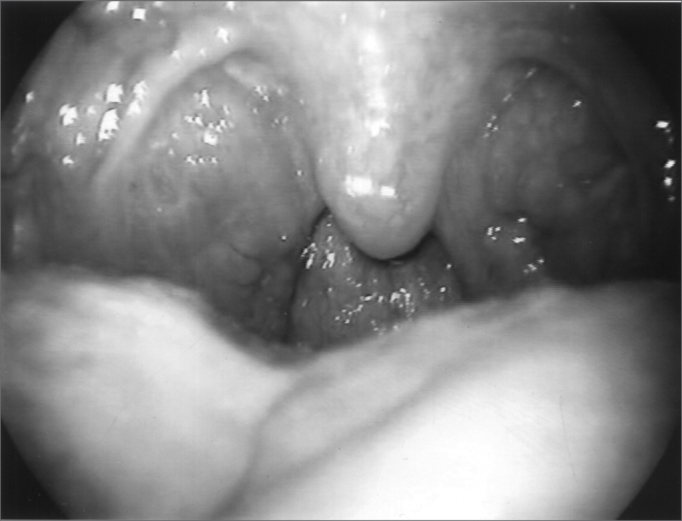
Pharynx after Botox injection during inspiration

Since 6 months ago, he started complaining of palate closure during nasal breathing (Figures 6 and 7) and showing dystonic palate movements at the nasolaryngoscopic evaluation. We then decided to inject Botulin toxin in the palate tensor veli palati muscle (10 UI), bilaterally, with symptoms improvement, although in the first month he complained of ear fullness. During the five months that followed, he remained asymptomatic and without complaints, even having a mild hyper nasality - consequent to Botulin toxin in the palate and blowing voice because of the laryngeal injection of BT. Symptoms recurred one month ago, and through nasofibroscopy we observed vocal fold paradoxical movements, palate and pharyngeal posterior and lateral walls movement during breathing. There were three levels of obstruction during inspiration: palate, pharynx and glottis. Figures 8 and 9 show the paralyzed palate after Botulin toxin injection and the pharynx at rest and contracted (also in an image through the mouth as seen in figures 10 and 11), with inspiration restriction. With this, in order to control dystonia, it was necessary to inject Botulin toxin in the TA and CAL muscles, the tensor veli and lateral pharyngeal walls bilaterally (10UI) (Figure 12). The patient is currently with a slight hyper nasality and blowing voice.

As to his daily activities, when under the Botulin toxin effect, the patient leads a normal life, without professional restrictions - he works in an office and does not have to talk to people. Although his voice is blowing and less intense, he does not complain because his major discomfort was only related to breathing. Even after pharyngeal injection, he does not have complaints related to swallowing. He practices sports such as squash and weight lifting, and only when contractions increase much at the end of the toxin effect, he feels a little out of breath during the exercises.

As to his past, he reports having had a calm childhood. He had many episodes of sinusitis, allergic rhinitis, tonsillitis and pharyngitis. He has had chicken pox and rubella. He does not smoke or drink alcohol. He does not have neck muscle pain, even when the contractions are intense. His cerebral MRI is normal and his polysomnography of August 2004 did not detect oxygen desaturation during sleep, and his lowest O_2_ saturation index was of 94%.

The patient also reports that his contractions are continuous and happen with a frequency of one each two seconds, and when he breathes deeply, the spasm is more intense, taking longer to start a new breathing cycle.

## DISCUSSION

Since dystonia is a disease of unknown etiology, its treatment is symptom-oriented.

In the hereby described case, the patient has been followed for almost five years, being diagnosed right at the onset of manifestations, when he was 19 years old.

The clinical manifestations coincide with literature descriptions, since we notice a worsening in symptoms in young patients. However, we did not find in the literature reports of palate and pharynx involvement following the paradoxical laryngeal movement.^4^,^5^ The spasms-caused movements impacted the patient's inspiration, and consequently, his life, for it caused a total closure of airway on the palate, pharynx or larynx, after inspiration onset. Thus, aware of treatment side effects, the patient allowed the injections that improved his breathing and worsen his phonation. He was pleased with his breathing improvement, which really bothered him, especially during physical activities. He reported no difficulties, except for blowing voice that did not bother him. The Botulin toxin dose should be the lowest possible; however, enough to control the symptoms, and thus cause less side effects and prevent the human body to develop antibodies against the toxin, which reduces its therapeutic effect^4^,^6^. We used the dose of 10 UI for each muscle injected.

## CONCLUSION

The lack of knowledge regarding etiology and the consequent lack of a proper therapy make it very difficult to treat dystonia. Botulin toxin allows us to control the contractions and its use in respiratory dystonia must consider the muscles affected, aiming at freeing the respiratory tract to inspiration with the least side effects.
